# Psychiatric and neurological disorders in late adolescence and risk of convictions for violent crime in men

**DOI:** 10.1186/s12888-015-0683-7

**Published:** 2015-11-23

**Authors:** Tomas Moberg, Marlene Stenbacka, Anders Tengström, Erik G. Jönsson, Peter Nordström, Jussi Jokinen

**Affiliations:** Department of Clinical Neuroscience/Psychiatry, Karolinska Institutet, Stockholm, Sweden; NORMENT, KG Jebsen Centre for Psychosis Research, Institute of Clinical Medicine, University of Oslo, Oslo, Norway; Department of Clinical Sciences, Psychiatry, Umeå University, Umeå, Sweden; Department of Clinical Neuroscience/Psychiatry, Karolinska Institutet, Karolinska University Hospital/Solna, SE-171 76 Stockholm, Sweden

**Keywords:** Mental retardation, Substance-related disorders, Personality disorders, Mental disorders, Conduct disorder, Childhood maltreatment, Violence and Violent crime

## Abstract

**Background:**

The relationship between mental illness and violent crime is complex because of the involvement of many other confounding risk factors. In the present study, we analysed psychiatric and neurological disorders in relation to the risk of convictions for violent crime, taking into account early behavioural and socio-economic risk factors.

**Methods:**

The study population consisted of 49,398 Swedish men, who were thoroughly assessed at conscription for compulsory military service during the years 1969–1970 and followed in national crime registers up to 2006. Five diagnostic groups were analysed: anxiety-depression/neuroses, personality disorders, substance-related disorders, mental retardation and neurological conditions. In addition, eight confounders measured at conscription and based on the literature on violence risk assessment, were added to the analyses. The relative risks of convictions for violent crime during 35 years after conscription were examined in relation to psychiatric diagnoses and other risk factors at conscription, as measured by odds ratios (ORs) and confidence intervals (CIs) from bivariate and multivariate logistic regression analyses.

**Results:**

In the bivariate analyses there was a significant association between receiving a psychiatric diagnosis at conscription and a future conviction for violent crime (OR = 3.83, 95 % CI = 3.47–4.22), whereas no significant association between neurological conditions and future violent crime (OR = 1.03, 95 % CI = 0.48–2.21) was found. In the fully adjusted multivariate logistic regression model, mental retardation had the strongest association with future violent crime (OR = 3.60, 95 % CI = 2.73–4.75), followed by substance-related disorders (OR = 2.81, 95 % CI = 2.18–3.62), personality disorders (OR = 2.66, 95 % CI = 2.21–3.19) and anxiety-depression (OR = 1.29, 95 % CI = 1.07–1.55). Among the other risk factors, early behavioural problem had the strongest association with convictions for violent crime.

**Conclusions:**

Mental retardation, substance-related disorders, personality disorders and early behavioural problems are important predictors of convictions for violent crime in men.

**Electronic supplementary material:**

The online version of this article (doi:10.1186/s12888-015-0683-7) contains supplementary material, which is available to authorized users.

## Background

The relationship between mental illness and violent crime in adults is complex owing to the involvement of many early risk factors. Psychotic disorders were over-represented in a well-characterised cohort of homicide offenders [1], as well as in studies on other violent crimes [2, 3] and other factors, such as substance use disorders, have been suggested to be a possible mediator of the increase in the risk of violent crime among patients with psychoses [4, 5]. With regard to other mental disorders, e.g., affective disorders, it has been a subject of controversy as to whether it is the affective disorder itself or other factors that contribute to the increased risk of violent crime. A recent study demonstrated a relationship between depression and violent crime [6], while some studies argue that this association is better explained by co-morbid substance abuse [5, 7] and is stronger for bipolar disorder [8–10]. Personality disorders and substance use disorders are the psychiatric conditions suggested to increase the risk of recurrence of violent crimes the most [11]. Antisocial personality disorder and borderline personality disorder, in particular, are considered to enhance the risk of violence [12], but violent behaviours, either directly or indirectly, constitute part of the operational criteria for these diagnoses and suggest caution in the interpretation of the literature [13]. Alcoholism and drug dependence are well-established risk factors for violent offending, and suggested factors for increasing the risk of violence in connection with substance use disorders are antisocial traits and behaviour [14] and a propensity to react with aggression in conflicts [15].

The impact of mental retardation on violent offending has been demonstrated in studies on inmates in the criminal justice system, where the prevalence of mental retardation has been reported to vary from 2 up to 40 %, the wide range being due to methodological factors [16]. Mental retardation was found to be an important risk factor for violence in an early longitudinal study [17], a result which, to some extent, has subsequently been disputed [18]. Mental retardation is not, however, included as a primary risk factor for violence in violence risk assessment instruments [19–23] even though some of the listed risk factors are likely to be secondary manifestations of mental retardation, i.e., a lack of coping skills. Neurological disorders, e.g., epilepsy, and their significance for violent crimes have been very much in focus and of great interest in the history of forensic psychiatry [24], but studies have failed to demonstrate a connection with violent crimes [25].

Episodes of violence in adulthood are often preceded by behaviour problems at a young age [26]. Youths with an early onset of such problems are typically diagnosed with conduct disorder, which, independently [27] or in combination with severe mental illness [28], has been shown to pose an increase in the risk for violence in adulthood [29]. In addition, it has been suggested that there is a connection between childhood maltreatment and adult violence according to the ‘cycle of violence’ hypothesis [30]. Other similar confounders such as negative life events and low levels of social support might also explain an increased risk of violent offending among people with common mental disorders [31]. There is a paucity of long-term, longitudinal population-based follow-up studies that simultaneously examine psychiatric disorders with an early age of onset and mental retardation, as well as neurological disorders in relation to the risk of a later conviction for violent crime, while taking into account known early behavioural and socio-economic risk factors derived from widely used violence risk assessment instruments.

### Aims of the study

The main objective of this study was to investigate the impact of psychiatric diagnoses in late adolescence on convictions for violent crime later in life. A further aim was to investigate the significance of neurological disorders, as well as other early risk factors, in relation to convictions for violent crime.

Research questions:

1. Do psychiatric diagnoses in late adolescence increase the risk of a future conviction for violent crime?
2. Do neurological disorders increase the risk of a conviction for violent crime?
3. Which psychiatric diagnoses are most important for a future conviction for violent crime and what is the difference in their impacts?
4. To what extent are other risk factors in late adolescence relevant to a conviction for violent crime later in life?
5. Which diagnoses and other risk factors are of importance for relapse in convictions for violent crime?

## Methods

### Participants

The study population consisted of 49,398 Swedish men, born 1949–1951, who were assessed from 1 July 1969 to 30 June 1970, i.e., at an age of 18–20 years, in connection with the nationwide conscription for compulsory military service. The cohort consisted of 97–98 % of all possible conscripts in Sweden at that time. The remaining young men were exempted from conscription for such medical reasons as severe physical or psychiatric disorder/disability. The follow-up time for the survivors in the cohort was 35.24 years, standard deviation (SD) = 3.98. Conviction data were linked at Statistics Sweden via the unique Swedish civic registration number for each individual in the cohort. This number was then replaced with an individual serial number, making the data anonymous to the researchers, after approval by the Karolinska Institute Research Ethics Committee in Stockholm (Dnr 2007/ 174–31, Dnr 2008/1086-31/5). Using this procedure to guarantee the anonymity of data, the participants did not have to sign their informed consent [32]. The ethics committee was aware of that we were not going to obtain consent from participants when they approved the study.

The conscription procedure was a 2-day event, including physical tests and thorough examination by a physician to establish a physical diagnosis. Cognitive tests were performed and a psychologist interviewed all conscripts. A suspected psychiatric disorder resulted in referral to a psychiatrist for a clinical examination and mental disorders were recorded in accordance with the International Classification of Disease, Revision 8 (ICD-8) [33]. Only main diagnoses were included in the study. Main diagnoses were defined as the most clinically relevant diagnoses determined at conscription according to ICD 8. Details of this procedure and the validity of the assessments have been described earlier [34, 35]. The psychiatric conditions in the cohort were recorded in five different diagnostic groups according to the ICD-8 classification. Psychoses included diagnoses 292 and 295–298, anxiety-depression/neuroses 300, personality disorders 301, substance-related disorders 303 and 304, and mental retardation 310–315. A sixth group with neurological disorders was created by grouping the following diagnoses: 309, 320, 321, 323, 324, 330, 332, 343–345 and 348. The diagnostic numbers from the ICD-8 classification are presented in Additional file 1.

In the total cohort of 49,398 conscripts, 9.90 % (*n* = 4892) were diagnosed with a psychiatric or neurological condition as defined above. The largest diagnostic group was anxiety-depression/neuroses with a prevalence of 4.90 % (*n* = 2419) in the cohort, followed by personality disorders with 2.56 % (*n* = 1267). Mental retardation had a prevalence of 1.06 % (*n* = 524) and was slightly higher than substance-related disorders with 0.93 % (*n* = 459) at conscription. The smallest groups were neurological disorders and psychoses with 0.39 % (*n* = 192) and 0.063 % (*n* = 31), respectively.

#### Other potential risk factors (confounders)

Based on the literature on violence risk assessment, additional risk factors (confounders) were selected from the conscription data using the following procedure. Five risk assessment instruments, i.e., the Level of Service Inventory-Revised (LSI-R) [19], the Psychopathy Checklist-Revised (PCL-R) [20], the Historical-Clinical-Risk Management-20, Version 3 (HCR-20^V3^) [21], the Spousal Assault Risk Assessment: Short Version (SARA: SV) [22] and the Violence Risk Screening-10 (V-RISK-10) [23], were scrutinised and 124 variables were clustered into 21 domains. Conscription data obtained from 105 questions in two self-report questionnaires were assessed. Thirty-one questions contained information that corresponded to 30 variables in 11 domains in the violence risk assessment instruments and which were statistically significantly associated with risk of future convictions for violent crime and were therefore chosen as potential confounders/risk factors. The question/variable from each domain that was most significantly associated with a conviction for violent crime was selected; only three questions were not included in the study because of there being only a weak association. The following eight items from the conscription questionnaires were used for further analyses: Poor economic conditions in the family, Divorced parents, Corporal punishment in upbringing, Easily angry, Sleep disturbance (i.e., reflecting stress), Lowered marks due to misconduct at school, Contact with the police or child welfare section of a municipal welfare committee (referral to the child welfare section was due to either the child’s misconduct problems or maltreatment in the family) and Arrested by police for drunkenness. The last of these risk factors was not excluded despite the fact that substance-related disorders were among the analysed diagnoses because the variable itself was considered to reflect a certain type of behaviour not always caught in a dependence diagnosis. The following domains of risk factors derived from the literature on risk assessment instruments did not have corresponding information in the conscription data: Criminal history as an adult, Bad conduct in prison or under probation, Unemployment, Economic problems as an adult, Marital problems or promiscuous sexual behaviour, Living in an area with high criminality, Having friends with criminal history, Negative attitudes toward legal systems, or a rating for Psychopathy or lack of empathy.

#### Crime

Through the conscripts’ unique Swedish civic registration numbers, the cohort was linked by record to the National Crime Register. The Swedish National Council for Crime Prevention, an agency under the Ministry of Justice and a centre for research and development within the judicial system, granted permission to access the conviction data, which were obtained to identify the date, type and number of criminal offences during the years 1971–2006. The studied outcome measure was a conviction for a violent crime (*n* = 2532), which was defined as homicide (*n* = 22), manslaughter (*n* = 135), aggravated assault (*n* =162), assault and battery (*n* = 2102), bodily harm (*n* = 107) and other (*n* = 4). Sex crimes and robbery were not included in the category of violent convictions. No violent crime was defined as all other convictions or a lack of any conviction. We also categorised offences according to the number of convictions for violent crime (one versus two or more).

### Statistical analyses

The relative risks of convictions for violent crime during the years 1970–2006 were examined in relation to psychiatric and neurological diagnoses and other risk factors at conscription during 1 July 1969 to 30 June 1970, as measured by odds ratios (ORs) with 95 % confidence intervals (CIs) from bivariate and multivariate logistic regression analyses (SAS 9.1, SAS Institute Inc., Cary, NC, USA). The outcome measure was any conviction for violent crime. In the bivariate analyses, the reference categories comprised all individuals without any psychiatric or neurological diagnosis or without any other risk factor at conscription, with the independent variables being measured only once. Only significant diagnostic and other risk factor variables in the bivariate analyses were included in the multivariate analyses. Potential confounders/other risk factors for the bivariate and multivariate analyses were selected by testing for all other possible risk factors using χ2 tests (categorical variables) and retaining the one in each domain that was the most significant predictor for conviction for a violent crime. Tests for non-parametric correlations using Spearman’s rho verified that the risk factors (i.e., ordinal variables from questionnaires) did not correlate with each other. Group differences concerning age of onset of violent disorder convictions were tested with the Kruskal-Wallis test. The *p*-value was set at < 0.05 in all the analyses.

## Results

### Psychiatric diagnoses and future convictions for violent crime

Fifteen per cent of the men with a psychiatric diagnosis at conscription had been convicted for at least one violent crime during the follow-up period, compared to 3.7 % of the conscripts without a psychiatric diagnosis at conscription. There was a significant association between receiving a psychiatric diagnosis at conscription and a future conviction for violent crime (odds ratio (OR) 3.83, 95 % confidence interval (CI) 3.47–4.22). The analysis of specific psychiatric diagnostic groups revealed that substance-related disorders had the strongest association with convictions for violent crime (OR 10.08, 95 % CI 8.18–12.42), followed by mental retardation (OR 5.65, 95 % CI 4.50–7.09) and personality disorders (OR 5.36, 95 % CI 4.60–6.25). Anxiety-depression (OR 1.99, 95 % CI 1.69–2.34) and psychosis (3.83, 95 % CI 1.34–10.96) had a weaker association with violent crime conviction.

### Neurological conditions in relation to future convictions for violent crime

Among persons with neurological diagnoses, 3.7 % were convicted for violent crimes during the follow-up. There was no significant association between neurological conditions and future convictions for violent crime (OR 1.03, 95 % CI 0.48–2.21).

### Other risk factors relevant for future conviction for violent crime

Table 1 shows bivariate logistic regression analyses between selected other potential risk factors measured at conscription and future convictions for violent crime. Arrested by police for drunkenness had the strongest association with future convictions for violent crime (OR 5.91, 95 % CI 5.36–6.50), followed by Contact with the police or a child welfare committee (OR 5.32, 95 % CI 4.87–5.82).

**Table 1:** Adolescent risk factors for future convictions for violent crime

Variables	Violent crime
	OR (95 % CI)
Poor economic conditions in family (very or rather poor vs. average, rather or very good)	1.42 (1.24–1.64)
Divorced parents (yes vs. no)	2.87 (2.60–3.18)
Corporal punishment in upbringing (often or sometimes vs. seldom or never)	1.96 (1.76–2.18)
Easily angry (often vs. sometimes, seldom or never)	3.13 (2.80–3.51)
Sleep disturbance (often vs. sometimes, seldom or never)	1.76 (1.52–2.04)
Lowered marks due to misconduct at school (several times or once vs. never)	3.92 (3.61–4.27)
Contact with the police or child welfare committee (several times or sometimes vs. never)	5.32 (4.87–5.82)
Arrested by police for drunkenness (several times, twice or once vs. never)	5.91 (5.36–6.50)

### Multivariate regression analyses

Table 2 shows the multivariate analyses in Models I–IV. Individuals meeting the diagnostic criteria for psychosis (*n* = 31) were not included in the models due to the small number for multivariate analyses. The remaining four groups of psychiatric diagnoses were included (Model I) with successive addition and stepwise adjustment with other potential risk factors in upbringing conditions (Model II), personal factors (Model III) and early behavioural problem variables (Model IV), respectively. Of all the diagnoses, substance-related disorders had the strongest association with future convictions for violent crime in Model I–III, followed by mental retardation and personality disorders. In the fully adjusted model, mental retardation had the strongest association with future convictions for violent crime (OR 3.60, 95 % CI 2.73–4.75), followed by substance-related disorders (OR 2.81, 95 % CI 2.18–3.62) and personality disorders (OR 2.66, 95 % CI 2.21–3.19), while anxiety-depression only had a weak, but significant, association (OR 1.29, 95 % CI 1.07–1.55).

**Table 2:** Multivariate analyses for future convictions for violent crime in Models I–IV

	Model I	Model II	Model III	Model IV
	OR (95 % CI)	OR (95 % CI)	OR (95 % CI)	OR (95 % CI)
Anxiety-depression (yes vs. no)	1.99 (1.69–2.34)	1.79 (1.51–2.12)	1.63 (1.37–1.94)	1.29 (1.07–1.55)
Personality disorder (yes vs. no)	5.36 (4.60–6.25)	4.90 (4.16–5.76)	4.29 (3.62–5.08)	2.66 (2.21–3.19)
Substance-related disorder (yes vs. no)	10.08 (8.18–12.42)	8.18 (6.53–10.26)	7.55 (5.98–9.54)	2.81 (2.18–3.62)
Mental retardation (yes vs. no)	5.65 (4.50–7.09)	4.95 (3.87–6.33)	4.39 (3.40–5.67)	3.60 (2.73–4.75)
Poor economic conditions in family		1.00 (0.86–1.17)	0.98 (0.84–1.15)	1.00 (0.85–1.18)
(very or rather poor vs. average, rather or very good)				
Divorced parents		2.32 (2.08–2.59)	2.30 (2.06–2.58)	1.68 (1.49–1.90)
(yes vs. no)				
Corporal punishment in upbringing		1.61 (1.43–1.80)	1.52 (1.35–1.71)	1.29 (1.14–1.46)
(often or sometimes vs. seldom or never)				
Easily angry (often vs. sometimes, seldom or never)			2.15 (1.89–2.45)	1.72 (1.50–1.98)
Sleep disturbance (often vs. sometimes, seldom or never)			0.93 (0.78–1.11)	0.84 (0.70–1.01)
Lowered marks due to misconduct at school (several times or once vs. never)				2.11 (1.91–2.34)
Contact with the police or child welfare committee (several times or sometimes vs. never)				2.67 (2.38–2.99)
Arrested by police for drunkenness (several times, twice or once vs. never)				2.06 (1.82–2.32)

Model 1. Model fit: Chi-square = 798.98, DF = 4, *p* < 0.0001; *p*-values of all included predictors < 0.0001

Model 2. Model fit: Chi-square =1032.38, DF = 7, *p* < 0.0001; p-values of all included predictors except poor economic conditions in family (*p* = 1.00) were significant, *p* < 0.0001

Model 3. Model fit: Chi-square = 1128.36, DF = 9, *p* < 0.0001; *p*-values of all included predictors except poor economic conditions in family (*p* = 0.80) and sleep disturbance (*p* = 0.44) were significant, *p* < 0.0001

Model 4. Model fit: Chi-square = 2231.10, DF = 12, *p* < 0.0001; *p*-values of all included predictors except poor economic conditions in family (*p* = 0.98) and sleep disturbance (*p* = 0.058) were significant: Anxiety-depression, *p* = 0.0074, all other *p* values < 0.0001

Considering other risk factors than psychiatric diagnoses in the fully adjusted model, early behavioural problem variables had the strongest association with future convictions for violent crime. The variables Divorced parents, Corporal punishment in upbringing and Easily angry were also significantly associated with a future conviction for violent crime, but to a lesser extent, whereas Poor economic conditions in family and Sleep disturbance did not show any significant association with future convictions for violent crime in this study.

### Onset of and relapse in violent crimes

The mean (standard deviation [SD]) age of onset for a future conviction for violent crime after conscription in the group with no psychiatric diagnoses was 26.5 (8.1) years. The mean age of onset was significantly lower for substance-related disorders, personality disorders and mental retardation with 21.5 (3.6), 21.9 (4.7) and 23.4 (5.7) years, respectively, compared to the group with no psychiatric diagnoses (*p* < 0.001). The age of onset for conscripts with anxiety-depression diagnoses was 25.9 (8.1), which is not statistically significantly different (*p* = 0.0825). The regression analysis was repeated in a fully adjusted multivariate model with the outcome of at least one violent crime relapse. The results of this analysis showed that mental retardation (OR 4.02, 95 % CI 2.60–6.22), personality disorder (OR 3.67, 95 % CI 2.81–4.79) and substance abuse (OR 2.99, 95 % CI 2.06–4.32) groups had significantly higher risks of violent crime relapse compared to the depression/anxiety group (OR 1.40, 95 % CI 1.03–1.89). There was also a stronger association with recidivism in violent crime for the other relevant risk factors; this was particularly evident for early behavioural problem variables.

## Discussion

The main finding in this long-term follow-up study was that men with a diagnosis of mental retardation at conscription had the highest risk for a future conviction for violent crime, taking into account other early risk factors. This result is in line with longitudinal studies showing that low intelligence is a significant risk factor for offending [17, 36–38]. In our study, mental retardation had a stronger association with convictions for violent crime than psychoses, anxiety-depression/neuroses, personality disorders and substance-related disorders. However, this is not reflected in any of the examined violence risk assessment instruments that do not directly take into account mental retardation as a diagnosis, but, instead, several of the other psychiatric diagnoses or risk factors. When interpreting our results it should be noted that the military authorities had excluded individuals considered not fit for military service before conscription. Among others, this group also included individuals with more severe forms of mental retardation requiring support from health and social services. In the present study, we also chose to include individuals diagnosed with borderline mental retardation (intelligence quotient 70–85) as mentally retarded because this group is well-represented in clinical populations when doing violence risk assessments. Other researchers have also called attention to this group’s inclusion in earlier studies on criminal careers [18]. This study had information on confounding factors considered relevant in a risk assessment perspective, thus enabling adjustment for different behavioural variables most likely not linked to mental retardation *per se*, but, instead, indicating a more general propensity towards anti-social behaviour/attitudes due to other conditions or circumstances. Poor coping skills due to mental retardation may sometimes be misinterpreted to be an anti-social behaviour caused by a conduct disorder. Based on our main findings, we recommend that individuals with an early onset of violent crime should, in appropriate cases, undergo a thorough neuropsychiatric investigation to determine whether there is masked mental retardation. The earlier the detection, the better the basis there is for planning appropriate support adjusted to the mental retardation, which may lead to a lower risk of violence.

Not surprisingly, substance-related disorders were associated with an increased risk of future convictions for violent crime in line with what other studies have shown [39, 40]. The result was robust even after adjusting for potential confounders, some of them corresponding to antisocial personality traits. This, together with the design of the study with an investigation of a birth cohort and thus excluding enrichment of antisocial traits through a selection bias, allowed us to make a realistic estimation of the extent to which the substance related disorder itself is a predictor of a violent crime. On the other hand, in our cohort, substance-related disorders consisted mainly of drug dependency (67 %), i.e., illegal substances connected with a high acceptance of anti-social acts. In reality, however, alcoholism is far more prevalent in the Swedish citizenry and it could be speculated whether a truer representation (better detection) of substance use problems in the cohort would alter the findings and, if so, in what direction.

A personality disorder diagnosis at conscription was also associated with an increased risk for a later conviction for violent crime in our study. This is in accord with a large-scale review which showed that all personality disorders evinced an increase in the risk for violent crimes [41]. In our statistical analyses, we handled the personality disorders as a single category because other and unspecified personality disorders accounted for most individuals in the group. The association between personality disorders and convictions for violent crime could not be explained by a high proportion of individuals with an antisocial personality disorder, who only accounted for 7 % (*n* = 89 out of 1267) of the personality disorders. The importance of personality disorders for conviction for violent crime remained significant after adjusting for factors that reflect early behavioural problems. In fact, deviant behaviour variables were often connected with personality disorders.

In our study, affective and anxiety disorders diagnosed at conscription were associated with a conviction for violent crime later in life, but, to a great extent, the association could be explained by other factors. Nevertheless, affective and anxiety disorders still remained a weak, but significant, predictor of a future conviction for violent crime even after adjusting for various potential confounders. This result is in line with what other authors have reported [42, 43]. In our study, the ICD-8 diagnoses were based upon the neuroses concept, clustering together affective disorders and anxiety disorders in a single group. We chose to keep that diagnostic grouping in our analyses in order to do justice to the diagnostic procedure during the years 1969–1970. However, during those years, bipolar disorder was diagnosed as a psychotic syndrome according to ICD-8 when manifested as an affective psychosis, manic or depressed type, thus accounting for over a third of the psychosis group (*n* = 13 out of 31). There were probably also some depressed conscripts with a masked bipolar disorder because they had not yet developed a manic episode. Affective disorders, especially depression, are common conditions often having an onset later in life and therefore it was not possible to include them in our analyses. An earlier study has also demonstrated considerable heterogeneity in the affective group with a higher criminality rate in bipolar patients and patients suffering from unipolar minor or intermittent depression, and no increased criminality rate in patients with unipolar major depression [44].

There was an association between psychosis and convictions for violent crime in the bivariate analysis of our study. Due to the small number of individuals with psychosis diagnoses, we could not determine whether the association between psychosis at conscription and a future conviction for violent crime later in life was due to other factors, such as personality pathology or co-morbid substance abuse, as suggested in the literature [4, 45]. It has been shown in a previous study that only a minority of the possible psychosis cases could be diagnosed at the time of conscription, since more than 90 % of the conscripts had their first-episode of psychosis later in life [46]. However, violent crimes often occur before clear psychotic symptoms appear [47, 48], thus probably resulting in an underestimation of the problem of conviction for violent crime in the psychosis group in our study.

Among the other eight confounding risk factors adjusted for, early behaviour problems had the strongest association with convictions for violent crime in adulthood. In particular, the variable ‘Contact with the police or child welfare committee’, but also ‘Lowered marks due to misconduct at school’ and ‘Arrested by police for drunkenness’, were associated with a conviction for violent crime. Most probably, the self-reported information in this context reflects an expression of some kind of aggressive behaviour or similar misbehaviour during childhood or adolescence. Our result was therefore expected and in line with previous research [26, 49]. Offending in childhood (whether violent or otherwise) is a key risk factor for persistent offending [26]. In our study, very few conscripts (*n* = 195 out of 49,398) had a violent offence conviction (according to criminal records) prior to the age of 18. Since this group was small, adding it to the multivariate model instead of ‘Contact with the police or child welfare committee’ did not change the results concerning the risk of convictions for violent crime and psychiatric diagnoses, we decided to use the broader construct in accordance with most violence risk assessment instruments.

To have been exposed to violence during childhood in the form of corporal punishment had an association with convictions for violent crime, but the relationship was weaker compared to have had divorced parents. In the present study, there was thus a weak connection between childhood maltreatment and violent conviction, giving some support to the ‘cycle of violence’ hypothesis along with previous studies [30, 50]. In summary, after adjusting for eight carefully selected confounders, the main finding remained robust, i.e., all studied psychiatric conditions, except psychoses, diagnosed at enrolment were associated with a conviction for violent crime later in life.

The strength of this longitudinal study includes the fact that the conscripts were from a large birth cohort recruited nationwide with a coverage of 97–98 %, who were thoroughly examined during 2 days by psychologists, physicians and, if necessary, psychiatrists in a procedure that included cognitive tests. Furthermore, the studied outcome, conviction for violent crime after conscription, was obtained from national crime registers covering a period of more than 35 years. The mean age at onset was thus a measure of offending in adulthood, because convictions for violent crime before conscription were not included.

A limitation is that women were exempted from conscription at that time, which restrains the generalisability of the findings of psychiatric diagnoses and future convictions for violent crime only to men. Furthermore, we did not have information about Attention deficit hyperactivity disorder and too few subjects with traumatic brain injury to analyse these conditions. In this study, the category violent crime conviction was based on the Swedish Penal Code categorisation of violent crime. Sex crimes and robbery were not included in the category which can be seen as a limitation. Detailed information on the adult experiences of the population that are likely to influence the offending trajectories, including the provision of support, medication and more detailed socio-economic data later in life, was not available. Some of the men with psychiatric disorders probably were not conscripted and we could only focus on those with a relatively early onset of psychiatric and neurological disorders. Thus, the sample does not represent the entire male population with these disorders.

## Conclusions

When performing violence risk assessments in men, it is important to do a thorough psychiatric and psychological evaluation, including cognitive tests, and to obtain information about early behavioural problems and childhood maltreatment. Mental retardation, substance-related disorders, personality disorders and early behavioural problems are important predictors of convictions for violent crime in men. Neurological disorders are not a predictor of convictions for violent crime in men. Poor coping skills due to mental retardation may sometimes be misinterpreted to be an anti-social behaviour caused by a conduct disorder. Individuals with an early onset of violent crime should, in appropriate cases, undergo a thorough neuropsychiatric investigation to determine whether there is masked mental retardation. The earlier the detection, the better the basis there is for planning appropriate support adjusted to the mental retardation, which may lead to a lower risk of violence. Based on our findings, we suggest considering inclusion of mental retardation in future revisions of the violence risk assessment instruments.

### Availability of supporting data

For data requests, please contact Marlene Stenbacka, Associate Professor. Data is not stored elsewhere due to confidentiality reasons in accordance with the decision from the ethics committee.

## Additional file

Additional file 1:
**Presentation of the diagnostic numbers from the ICD-8 classification used in this study.** (DOCX 68 kb)

## Abbreviations

CI: Confidence interval
ICD: International classification of diseases
OR: Odds ratio
SD: Standard deviation
