# Supplementary material for: Psychiatric and neurological disorders in late adolescence and risk of convictions for violent crime in men
Source: BMC Psychiatry. 2015 Nov 23;15:299. doi: 10.1186/s12888-015-0683-7 (PMC4657257; doi:10.1186/s12888-015-0683-7)
Supplement: Additional file 1: — Presentation of the diagnostic numbers from the ICD-8 classification used in this study. (DOCX 68 kb) [file 12888_2015_683_MOESM1_ESM.docx]

**Additional file 1:**

**Compilation of the diagnostic numbers from the International Classification of Disease, Revision 8 (ICD-8) used in the study ‘Psychiatric and neurological disorders in late adolescence and risk of convictions for violent crime in men’.**

**Psychoses**

292 Psychosis associated with intracranial infection

295 Schizophrenia

296 Affective psychoses

297 Paranoid states

298 Other psychoses

**Anxiety-depression/neuroses**

300 Neuroses

**Personality disorders**

301 Personality disorders

**Substance-related disorders**

303 Alcoholism

304 Drug dependence

**Mental retardation**

310 Borderline mental retardation

311 Mild mental retardation

312 Moderate mental retardation

313 Severe mental retardation

314 Profound mental retardation

315 Unspecified mental retardation

**Neurological and other central nervous system conditions**

309 Mental disorders not specified as psychotic associated with physical conditions

320 Meningitis

321 Phlebitis and thrombophlebitis of intracranial venous sinuses

323 Encephalitis, myelitis and encephalomyelitis

324 Late effects of intracranial abscess or pyogenic infection

330 Hereditary neuromuscular disorders

332 Hereditary ataxia

343 Cerebral spastic infantile paralysis

344 Other cerebral paralysis

345 Epilepsy

348 Motor neurone disease
